# Multidisciplinary Management Strategies for Long COVID: A Narrative Review

**DOI:** 10.7759/cureus.59478

**Published:** 2024-05-01

**Authors:** Christian Prusinski, Dan Yan, Johana Klasova, Kimberly H McVeigh, Sadia Z Shah, Olga P Fermo, Eva Kubrova, Ellen M Farr, Linus C Williams, Gerardo Gerardo-Manrique, Thomas F Bergquist, Si M Pham, Erica Engelberg-Cook, Joshua M Hare, Keith L March, Arnold I Caplan, Wenchun Qu

**Affiliations:** 1 Department of Pain Medicine, Mayo Clinic, Jacksonville, USA; 2 Department of Transplantation, Mayo Clinic, Jacksonville, USA; 3 Department of Neurology, Mayo Clinic, Jacksonville, USA; 4 Department of Physical Medicine and Rehabilitation, Mayo Clinic, Rochester, USA; 5 Department of Internal Medicine, Lahey Hospital & Medical Center, Burlington, USA; 6 Division of Gastroenterology and Hepatology, Mayo Clinic, Rochester, USA; 7 Department of Psychiatry and Psychology, Mayo Clinic, Rochester, USA; 8 Department of Cardiothoracic Surgery, Mayo Clinic, Jacksonville, USA; 9 Department of Medicine, Cardiovascular Division and the Interdisciplinary Stem Cell Institute, Miami, USA; 10 Division of Cardiovascular Medicine, Center for Regenerative Medicine, University of Florida, Gainesville, USA; 11 Department of Biology, Case Western Reserve University School of Medicine, Cleveland, USA; 12 Center for Regenerative Biotherapeutics, Mayo Clinic, Jacksonville, USA

**Keywords:** a narrative review for long covid management, a multidisciplinary approach for long covid, management strategies for long covid, severe acute respiratory syndrome coronavirus 2, post-acute sequelae sars-cov-2 infection

## Abstract

Severe acute respiratory syndrome coronavirus 2 (SARS-CoV-2) has caused millions of infections to date and has led to a worldwide pandemic. Most patients had a complete recovery from the acute infection, however, a large number of the affected individuals experienced symptoms that persisted more than 3 months after diagnosis. These symptoms most commonly include fatigue, memory difficulties, brain fog, dyspnea, cough, and other less common ones such as headache, chest pain, paresthesias, mood changes, muscle pain, and weakness, skin rashes, and cardiac, endocrine, renal and hepatic manifestations. The treatment of this syndrome remains challenging. A multidisciplinary approach to address combinations of symptoms affecting multiple organ systems has been widely adopted. This narrative review aims to bridge the gap surrounding the broad treatment approaches by providing an overview of multidisciplinary management strategies for the most common long COVID conditions.

## Introduction and background

The public health emergency of coronavirus disease 2019 (COVID 2019), caused by severe acute respiratory syndrome Coronavirus 2 (SARS-CoV-2), has led to over 774 million infections and 7 million deaths as of March 3, 2024 [1]. Three years after the initial outbreak, the world faces long-lasting sequelae among survivors, who oftentimes continue to have multisystemic symptoms. Common terms for this variable combination of persistent signs and symptoms include ‘long covid’, ‘post-COVID syndrome’, ‘chronic COVID syndrome’, long-haul COVID [2,3], and ‘post-acute sequelae of COVID-19’ (PASC) [4,5]. Post-acute COVID is generally defined as the persistence of symptoms more than 4 weeks after infection and up to three months post-infection [6], while long COVID is defined by the World Health Organization (WHO) as symptoms persistent 3 months after the initial SARS-CoV2 infection, with these symptoms lasting for at least 2 months and not attributed to an alternative diagnosis [7,8]. The term “long COVID” will be adopted throughout this review.

While acute COVID infection (symptoms <4 weeks after infection) primarily affects the respiratory tract, long COVID is a multisystemic illness encompassing more than 100 symptoms, including myalgic encephalomyelitis/chronic fatigue syndrome (ME/CFS), cognitive blunting (“brain fog”), dysautonomia, endothelial dysfunction, cough, shortness of breath, chest pain (‘lung burn’), paresthesias, irritability, sleep disturbances, headaches, muscle pain and weakness, skin rashes, and cardiac, endocrine, renal and hepatic manifestations. There is a significant variability in the incidence of these symptoms (5-80%), possibly due to differences in study populations, novelty of the condition, and inconsistent diagnostic criteria [9-11]. In a recent meta-analysis, the estimated prevalence of long COVID worldwide was 45% [12]. The most prevalent sequelae were fatigue (25.2%), dyspnea (18.2%), and impairment of daily activities (14.8%) [12]. Hospitalized patients and females are at higher risk of developing long COVID, though this may be dependent on the specific type of long COVID symptoms [13]. The leading hypothesis for persistent symptoms includes lingering virus presence with restricted viral replication and subsequent immune dysregulation [14,15], autoimmunity with molecular mimicry and T-cell dysregulation [15-18], latent viruses reactivation and dysmicrobiosis [19], endothelial dysfunction with associated microvascular microclots [20], autonomic dysregulation, and direct tissue damage with insufficient tissue repair [21-25].

There are no currently agreed-upon clinical guidelines for the treatment of long COVID, although the CDC has released some guidance for healthcare providers when managing post-COVID conditions [26]. With a high frequency of multi-organ involvement, a team approach has been adopted that includes primary care and many subspecialties, including cardiology, pulmonology, neurology, hematology, physical medicine and rehabilitation, neuropsychiatry/neuropsychology, physical therapy, occupational therapy, as well as integrative medicine [27]. This review aims to comprehensively summarize the multidisciplinary treatment strategies with a detailed description of management for specific organs.

## Review

Methods

A literature review was conducted across multiple databases, including MEDLINE(Ovid), PubMed, Web of Science, and Google Scholar, covering the period from the inception until November 2023. A diverse set of keywords, such as “long covid”, “post-COVID syndrome”, “chronic COVID syndrome”, “long-haul COVID”, “post-acute sequelae of COVID-19”, “management strategies”, “treatment”, and “medications”, was employed. No limitations were imposed regarding study designs. Articles were rigorously assessed for their relevance in discussing current management strategies or clinical investigations related to long COVID. This narrative review presents the existing literature, categorized based on management approaches targeting specific organs or aspects of the conditions.

Pulmonary long COVID

Lungs are one of the primary organs affected by COVID-19. Fatigue, dyspnea, chest pain, and cough were the most prevalent respiratory symptoms found in 52%, 37%, 16%, and 14% respectively, in survivors admitted with COVID-19, 3 months after discharge [28,29]. Female sex, COVID-19 disease severity, and the requirement for invasive and non-invasive ventilation are risk factors for the development of pulmonary long COVID. Pulmonary long COVID may lead to obstructive phenotype with small airway disease [30,31], restrictive phenotype, also known as long COVID pulmonary fibrosis [32], pulmonary vascular disease [33], or any combination of these. Pulmonary fibrosis, most resembling idiopathic pulmonary fibrosis (IPF), is likely present in 2-6% of patients who suffered from moderate COVID disease [34], and is the most severe form of COVID-19-related lung disease leading to significant morbidity and mortality. The phenotypes and severity of pulmonary long COVID determine the management strategies and need for involvement of sub-specialities like interstitial lung disease, pulmonary hypertension, and/or lung transplant.

Patients with persistent dyspnea have greater restriction on spirometry, reduced functional capacity, and increased exertional desaturation and dyspnea [35]. Chest CT is recommended for follow-up, both for assessment of small airway disease, as well as to follow up on interstitial lung disease and pulmonary vascular disease [17].

Pharmacological Treatment Strategies

Bronchodilators: The usefulness of bronchodilators in COVID-19 is uncertain. In a bronchodilator reversibility study of inhaled salmeterol 400μg on patients’ post-moderate to severe COVID-19, all patients demonstrated improvement of their forced expiratory volume in the forced expiratory volume (FEV1) and forced vital capacity (FVC) with maximum improvement in patients with a prior history of asthma, suggesting that bronchodilators should always be considered in pulmonary long COVID to improve functional capacity of patients [36].

Inhaled corticosteroids: There is currently little evidence studying the effect of inhaled corticosteroids for pulmonary long COVID. Beneficial effects of secondary restoration of impaired antiviral immunity were found in asthmatic patients with COVID-19 [37]. Future studies are warranted to evaluate the role of inhaled corticosteroids given these findings and the pathophysiology of small airway disease seen in pulmonary long COVID. 

Systemic corticosteroids: A recent guideline from the Swiss COVID Lung Study group and Swiss Society for Pulmonology (SSP) has recommended glucocorticoids for the treatment of post-COVID-19 diffuse parenchymal lung abnormalities [38,39]. A study showed clinical-physiologic improvement in patients with persistent clinical symptoms and organizing pneumonia patterns after treatment with medium-dose corticosteroids [39]. An open-label, randomized trial evaluated the effect of high dose (40 mg/daily for 1 week followed by 30 mg/daily for 1 week, followed by 20 mg/daily for 2 weeks, and subsequently 10 mg daily for 2 weeks) versus low dose (10 mg/daily for 6 weeks) prednisolone in symptomatic patients with post-COVID-19 diffuse parenchymal lung abnormalities [40]. Similar outcomes were found with improvement in clinical, radiologic, physiologic, and health-related quality of life (HRQoL) measures when comparing both doses on an average of five weeks after COVID-19 diagnosis [41].

Anti-fibrotic treatment: A case report from Japan suggested that Nintedanib may be a novel therapeutic for SARS-CoV-2 pulmonary fibrosis [42]. Pirfenidone is another drug that has been previously used in IPF disease and may be explored. According to the recent review, several clinical trials have been initiated to check the efficacy of certain antifibrotic drugs, including nintedanib, pirfenidone and a few others such as treamid and LYT-100 [39]. A few targets that may prove to be clinically relevant in future have recently been studied, including Lipocalin 2, MMP-7, and HGF [43].

Lung Transplantation

Select patients with COVID-related acute respiratory distress syndrome (CARDS) requiring extracorporeal membrane oxygenation (ECMO) for an extended period with no potential for recovery, and post-COVID-19 pulmonary fibrosis are being considered for lung transplantation [44]. It is crucial to ensure that appropriate time is allowed for the lungs to recover before patients are considered for lung transplant, especially in patients with organizing pneumonia who have the potential to recover over time, although this recovery may take months [45]. About 8.7% of lung transplants performed in the US between August 2020 and June 2022 were for COVID-related lung disease [3,46]. Whether this number will increase in the future is dependent on the emerging SARS-COV-2 variant characteristics and the effect of herd immunity. 

Cardiovascular long COVID

Multiple cardiac abnormalities were observed in patients recovering from COVID-19 after several weeks or even months, including left ventricle function, diastolic dysfunction, right ventricle dysfunction, or pericardial effusion or thickening [47,48]. The most common cardiovascular symptoms of long COVID are exercise intolerance, dyspnea, chest pain, and palpitations, with a prevalence of 29-34.5%, 15-22.3%, 6.7-7.0%, 4.5-6.0% respectively through 2 years post-infection [12,49,50]. Most cardiac symptoms alleviate with time, but some of them, especially diastolic dysfunction, may persist [51]. Also, the number and intensity of persistent cardiovascular symptoms correlated to the reduced quality of life(QoL) as reflected by transthoracic echocardiography, mental health examination, QoL questionnaire, and the Hospital Anxiety and Depression Scale [52]. Symptoms can be divided into two groups: long COVID with cardiovascular disease, and long COVID with cardiovascular symptoms without objective evidence of cardiovascular disease [53].

COVID-19 cardiovascular disease sequelae may be driven by myocardial involvement during the acute phase [54]. The possible origin of myocardial injury includes ischemic and non-ischemic processes e.g. pericarditis, myocarditis, acute coronary syndrome, endothelial dysfunction, nonischemic cardiomyopathy, thromboembolism, arrhythmia, and sequelae of chronic pulmonary disease [55,56]. Acute myocarditis in COVID-19 is probably less common than originally thought [54], but remains clinically relevant. Persistent local inflammation may result in dysrhythmias, fibrosis, and cardiomyopathy [57-59]. Treatment of cardiovascular disorder related to long COVID should be led by a cardiologist and follow the standard guidelines unrelated to COVID-19 infection [59]. Individuals with long COVID and cardiovascular disease can be referred to a cardiac rehabilitation program that promotes physical and mental improvements through structured lifestyle changes and exercise [60].

Cardiovascular symptoms without objective evidence of cardiovascular disease may present as tachycardia, palpitation, presyncope, exercise intolerance, and dizziness [61]. These may be triggered by dysautonomia or deconditioning after bed rest, producing orthostatic intolerance syndromes such as postural orthostatic tachycardia syndrome (POTS) [54,62]. The rehabilitation therapy should be symptom-titrated as tolerated [63]. For the treatment of moderate to severe symptoms, recumbent or semi-recumbent exercise at a submaximal level is recommended with slow progression to upright activities over time to avoid exacerbation of post-exertional malaise [54]. Other beneficial interventions consist of sufficient fluid and salt intake (5-10g/day), avoiding dehydration (alcohol and caffeine abstinence), optimization of chronic pharmacotherapy, behavioral modifications, and compression garments to reduce peripheral venous pooling and increase venous return [24,63]. Empirical pharmacology treatment can be considered if symptoms persist despite conservative measures. Individual case studies [64,65] and one clinical trial [66] have been conducted on the use of beta-blockers and ivabradine for patients with PASC-associated tachycardia and palpitations with variable outcomes. Other available medications include fludrocortisone to diminish the symptoms of hypovolemia, midodrine to increase vasoconstriction and venous return, clonidine [67], and methyldopa or propranolol to attenuate the hyperadrenergic syndromes [63].

The American College of Cardiology (ACC) has recently released a recommendation for professional athletes to return to play [54]. Further cardiac testing is recommended in athletes with persistent cardiovascular symptoms or those who require hospitalization with possible myocardial involvement. In asymptomatic athletes or athletes without cardiopulmonary symptoms, further testing is not recommended, and they can start with graded exercise with symptom reassessment every 24 hours [68]. If symptoms persist and cardiovascular disease is suspected, the training should be restricted followed by further cardiorespiratory evaluation according to COVID-19 unrelated guidelines.

Neurological long COVID

Headaches

One prospective cohort study of 100 patients with COVID-19 recovering from acute illness with long COVID reported neurological complaints in up to 59% of studied patients, with the most common being headaches [69]. Individuals with persistent headaches that may be SARS-CoV-2 related have been reported to be resistant to the typical prophylactic and abortive drug treatments used for headache treatments. It has been hypothesized that elevated cytokine levels may be potential contributors [70].

Pharmacologic management should nevertheless be directed by headache phenotype, as there are no proven superior treatments, akin to the treatment of new daily persistent headaches [71-73]. Headaches with tension features benefit from tricyclic antidepressants or serotonin-norepinephrine reuptake inhibitors (SNRI), trigger point injections, and physical therapy [74]. For migrainous headaches, standard migraine preventives should be tried first according to the guidelines published by the American Academy of Neurology and American Headache Society, which favor higher efficacy medications (divalproex sodium/sodium valproate, topiramate, beta-blockers, amitriptyline, and venlafaxine) [75]. Inability to tolerate, or inadequate response to therapeutic doses during an 8-week trial of two or more of the aforementioned drugs may be followed by a trial of monoclonal antibodies to the calcitonin gene-related peptide (CGRP) or its receptor (erenumab, galcanezumab, fremanezumab, and eptinezumab), or small molecule CGRP receptor antagonists (rimegepant and atogepant) [76]. Onabotulinumtoxin A injections can also be considered at that stage for chronic migraine. Medications should be introduced at low doses and in slow (weekly) titrations.

Non-pharmacologic interventions include pacing and taking “brain breaks” [77]. The goal is to avoid exacerbating symptoms through self-regulation of tasks and attention to aggravating symptoms. Tracking symptoms and slowly increasing the duration and intensity of cognitive tasks can help prevent headaches and reduce exacerbation. Examples include environmental management (such as reducing household stimuli, noise, and light) and encouraging single-tasking. It is important to counsel patients not to push through their symptoms, but to notice and pull back when symptoms arise. This technique is also used for the management of fatigue [77].

Neuropathy

Neurological problems following COVID-19 infection may be caused by several mechanisms including activation of innate or adaptive immunity or direct viral invasion [78]. Following a severe acute COVID-19 infection, a total of 59% patients were found to have neuropathy (most commonly small fiber neuropathy), and less frequently multifocal demyelinating neuropathy and critical illness axonal neuropathy [79-81]. Cranial nerve involvement during or following acute infection is also possible [82,83]. A cohort study of 1556 participants demonstrated that previously positive COVID-19 patients had a higher likelihood of neuropathy both post-acutely (within 90 days) as well as in the long COVID period [84]. Non-pharmacological treatments include rehabilitative interventions in the post-acute phase [31,85,86]. Symptoms of painful, small-fiber neuropathy were managed with common neuropathic agents such as tricyclic antidepressants (TCA), SNRIs, antiseizure medication pregabalin and gabapentin, and topical anesthetics [81]. Pharmacological treatment with intravenous immune globulins and/or corticosteroids was shown to provide incomplete recovery in up to 52% of patients treated in one study [80].

Stroke

A large case series and matched control study from Sweden showed that the risk for stroke two weeks after COVID-19 infection is about 6 times higher compared to controls. The stroke risk remains about twice as high in the 3rd and 4th week post-infection as compared to controls. Pharmacological and rehabilitative strategies in the long COVID patient population suffering from stroke will not differ from typical interventions for patients with stroke for the time being [87].

Coagulation disorders in long COVID

The high rate of thrombotic complications during the acute phase of COVID-19 has resulted in the development of recommendations for early anticoagulation [88-90]. However, no unified guidelines are provided for thromboprophylaxis in patients post-discharge. The incidence of thromboembolism in COVID-19 survivors at 30 days and 90 days is reported low as 0.4%-2.5% and 0.7-7.13% [89,91-96].

An American Society of Hematology (ASH) expert panel, supported by other guidelines, does not recommend the routine use of outpatient anticoagulant prophylaxis in patients with COVID-19 who are discharged from the hospital without suspected or confirmed venous thrombus embolism (VTE) or another indication for anticoagulation [89,97-100]. However, the evidence from multicenter prospective registry CORE-19 [92] reported a reduction in major thromboembolic events and all-cause death by 46% in high-risk patients receiving thromboprophylaxis with 10mg/day of rivaroxaban or prophylactic low-molecular-weight heparin (LMWH) [92]. Another multicenter, controlled trial (MICHELLE study) randomized 320 high-risk patients (IMPROVE VTE score >4 or IMPROVE VTE 2-3, and D- dimer 500ng/ml) to a reduced dose (10mg/day) of rivaroxaban for 35 days or no thromboprophylaxis [101]. The group with extended thromboprophylaxis manifested improved clinical outcomes (symptomatic venous or arterial thromboembolism, VTE-related death, bilateral VTE, myocardial infarction, non-hemorrhagic stroke, major adverse limb event, or cardiovascular death) without increasing the risk of major bleeding. An observational prospective study suggested a benefit of an extended (2-6 weeks) thromboprophylaxis with LMWH in high-risk patients (ICU stay, known thrombophilia, obesity, immobilization, heart failure, respiratory failure, age over 70 years, personal or familial history of VTE, active cancer or major surgery in the last 3 month) [102]. Based on low certainty in evidence for extended prophylaxis for post-COVID, individualized decisions should be made for each patient based on an assessment of thrombosis and bleeding risks.

One study of patients hospitalized for COVID-19 reported a successful restoration of endothelial function in 24 long COVID patients with a triple therapy (dual-antiplatelet therapy and anticoagulation). All patients reported exhaustion relief and brain fog resolution [103]. However, because of the significant risk of bleeding, this treatment is not recommended outside the research setting.

Due to limited evidence, large ongoing clinical trials (ACTIV-4, HEAL-COVID, CARE, CORONA-VTE NET, CISCO-19) are needed to warrant the role of continuing thromboprophylaxis, including antiplatelet therapy alone or in conjunction, in COVID-19 following discharge [104].

Neuropsychological long COVID

Insomnia

There are no current studies specifically evaluating treatment for sleep disturbances after COVID-19, however, sleep impairment is likely impacted by other symptoms [105]. Initially, discussing sleep hygiene and conservative management is most appropriate, along with ruling out or treating other confounding conditions such as obstructive sleep apnea, pain, anxiety, and depression [106]. Individuals with sleep disturbance may benefit from TCA drugs at bedtime. TCA therapy would also be of benefit for any underlying mood disorders [107]. For older patients, trazodone can be considered to improve sleep maintenance. There is no evidence for trazodone use in this population, however in populations of patients with dementia [108] or post-traumatic stress disorder [109], trazodone has been shown to be effective with a low side effect profile. 

Chronic Fatigue

Fatigue has been one of the most predominant symptoms of long COVID. A multi-faceted approach to fatigue may include a sleep questionnaire, Mallampati score, sleep hygiene, patient education information, a regimen of aerobic exercise and strength training with avoidance of graded exercise therapy [110], energy conservation, mindfulness, relaxation techniques, and medications [111]. Medications to treat fatigue in the multiple sclerosis (MS) population can be used in long COVID, with caution for increased tendency for side effects. It has not been reported to date that exposure to heat and humidity worsens fatigue in long COVID (as it does in multiple sclerosis) [112]. Patients may be queried in this regard and counseled appropriately when applicable. In addition, addressing and treating underlying or post-sequelae anxiety and depression can also impact fatigue symptoms.

Brain Fog

A recent systematic review of patients hospitalized for COVID-19 found studies that reported abnormal cognitive performance in 15.0-40.0% of study participants 10-105 days following hospital discharge [113]. The approach to cognitive dysfunction in these cases resembles that which is applied to persons with acquired brain injury. Recently, consensus guidelines from The American Academy of Physical Medicine and Rehabilitation (AAPM&R) were published for evaluation and treatment of long COVID-related cognitive impairment [114]. Screening instruments have been used to detect mental status changes in various etiologies in patients. Instruments with support in the literature for this purpose include the Montreal Cognitive Assessment (MoCA) [115], the Mini-Mental State Examination (MMSE) [116], the Saint Louis University Mental Status Examination (SLUMS), and the Short Test of Mental Status (STMS) [117]. Several of these tests have published norms that are more current, extensive, and stratified for age [118,119]. Given the known impact of aging on cognitive performance, anyone using such a screening instrument should be careful to use age-adjusted norms when conducting assessments with older adults.

Neuropsychological testing remains the “gold standard” for assessing metal status changes in medical and psychiatric parameters, as it is more extensive, more sensitive, and as a result more likely to detect changes in mental status than any of the mental status screening measures described above [120]. A recent study from Europe using neuropsychological assessment in individuals one-year post COVID-19 found 18% of the persons evaluated to have cognitive dysfunction. Additionally, cognitive dysfunction was found to be related to lower education, pre-infection history of headache/migraine, and the presence of headache and sleep disturbance at the time of acute infection [121]. For these reasons and as previously discussed, patients should be evaluated for other confounding conditions which may exacerbate cognitive impairment, such as poor sleep, mood disorders, endocrine abnormalities, and autoimmune disorders. While there is no literature in support of rehabilitative therapy for treating ‘brain fog’ in long COVID, there is a significant evidence base supporting the effectiveness of cognitive rehabilitation for impairments in multiple cognitive domains, including memory, attention, executive functioning, and visual spatial functioning, in conditions similar to long COVID such as myalgic encephalomyelitis/chronic fatigue syndrome [122,123]. Compensatory and functional strategies implemented during cognitive rehabilitation includes the incorporation of simple functional strategies in day-to-day life that assist in creating habits and routines, reducing brain fog, and improving recall. Other strategies include the utilization of activity logs, symptom diaries, calendars, and smart devices (Alexa, Google, and smartphones), which can be helpful in providing greater awareness, identifying changes in health or symptoms, and identifying potential triggers for fatigue [122,124].

Depression and post-traumatic stress

A previous study has demonstrated that the rates of a variety of mental health conditions were high in the weeks and months after discharge for COVID-19 related hospitalization [113]. Rates of depression were reported between 10% and 69% in one systematic review [113], while two meta-analysis [125,126] reported the depression range in COVID-19 survivors between 21-45%, anxiety 5% to 45-55% [113,126] and post-traumatic stress between 10% and 36.4% [113,127]. There is no evidence examining specific interventions to treat mood disorders in persons with long COVID. However, both disorders of mood and emotional symptoms secondary to medical conditions have been shown to be effectively treated by evidenced-based treatments such as cognitive behavioral therapy (CBT) [128,129], acceptance and commitment therapy (ACT) [130,131], aerobic exercise [132,133], and treatment of sleep disturbances [133,134].

Musculoskeletal long COVID

Muscle pain as well as joint pain have been reported in long COVID syndrome, specifically in 48% of patients in one cohort study of 100 patients [69]. Findings of myositis and elevated CK with suggestive biopsy were noted along with elevated autoinflammatory markers that are typically associated with rheumatological conditions [92,135,136].

Impaired musculoskeletal physical function and fitness have been reported to persist for 1-2 years following SARS-CoV-2 [137]. Patients with long COVID report significantly reduced physical function which is compounded by the cognitive and psychological effects of the illness [138]. This is likely due to a combination of musculoskeletal pain, deconditioning and cardiovascular sequela [139], muscle weakness [140], dysautonomia, and fatigue [24]. Musculoskeletal complaints may be treated with medications, physical therapy and occupational therapy [138], a guided exercise program, stretching techniques, yoga, relaxation exercises, application of moist heat or cold to affected areas, and alternative medicine techniques including osteopathic manual treatment (particularly indirect and counterstain techniques) [25] and acupuncture [141]. Tailored physical activity counseling is recommended [138]. Rehabilitation should employ a risk-stratification approach that emphasizes minimizing post-exertional malaise [142,143]. Graded exercise therapy that introduces physical activity gradually (often in the form of gentle stretching for a duration of as little as 5 min per day) may be used in patients with long COVID. Frenkel coordination exercises (FCE) were originally developed to treat patients with neurological ataxia and tabes dorsalis. These are a series of motions of increasing difficulty to facilitate the restoration of coordination intended to improve rhythmic, smooth, coordinated movements. FCEs have been shown to reliably reduce fatigue in the MS population and may be extended to treat fatigue related to long COVID [144].

Gastrointestinal long COVID

Enteric symptoms are common in COVID-19 and the gastrointestinal tract has been proposed as an entry route for COVID-19 virus through the ACE2 receptor in gastrointestinal mucosa [92]. The initial COVID-19 infection has been followed by prolonged viral fecal shedding [92,145] and alterations in the gut microbiome [126,146].

The most common symptoms at 3 months were loss of appetite (24%), nausea (18%), acid reflux (18%), and diarrhea (15%), followed by abdominal distension (14%), belching (10%), vomiting (9%), abdominal pain (7%), and bloody stools (2%) [147]. Another study reported abdominal pain (7.5%), constipation (6.8%), diarrhea (4.1%), and vomiting (4.1%) at a median follow-up of 106 days in hospitalized patients without a history of GI disease [148].

Gut microbiota dysbiosis was identified as a possible component of gastrointestinal long COVID pathophysiology [149]. Studies of the gut microbiome composition in patients with long COVID identified a change in various species, including *Faecalibacterium prausnitzii* [150,151]. One study has reported a positive effect of probiotic treatment on fatigue in long COVID, however, the clinical trials evaluating the effect of probiotic treatment on gastrointestinal symptoms are yet to be performed [152]. One randomized control trial from UK compared the treatment with a phytochemical-rich concentrated food capsule in addition to a pre/probiotic *Lactobacillus* capsule in patients post-acute COVID and with long COVID [80]. From the cohort of 147 patients, 31 patients reported having bowel symptoms at baseline which improved in 82% of participants after 30 days of treatment. Another case report demonstrated the alleviation of severe gastrointestinal symptoms in a patient with long COVID by treatment with ingestion of a high-fiber diet, which led to microbiome changes [146]. There are currently no validated effective treatments for gastrointestinal long COVID with only limited evidence available.

Renal long COVID

Acute renal injury occurs in about 28% of hospitalized COVID-19 patients likely through several mechanisms, with the most common pathophysiology being acute tubular necrosis like other non-COVID causes such as sepsis [153]. There have been signs of viral infiltration and local inflammation of the kidney, epithelial injury, and microthrombi affecting circulation. Collapsing glomerulopathy has been identified in patients with high-risk *APOL1* genotypes [154]. Fortunately, anti-inflammatory drugs have been shown to decrease renal injury [155]. Although most of the patients recover, there is a proportion of patients who develop chronic kidney disease (CKD), likely due to continued inflammation, tubular injury, and inadequate repair after acute kidney injury (AKI). These are commonly associated with comorbidities that further contribute to renal injury such as diabetes and hypertension [154,156]. No specific treatments for long COVID-related kidney dysfunction have been established.

Stem cells as a potential novel therapeutic strategy for long COVID

Regenerative medicine, specifically cell-based therapy with mesenchymal stem cells (MSCs), has been emerging as a potential novel treatment strategy for acute respiratory distress syndrome (ARDS) during the COVID-19 pandemic [51,99]. MSCs reside naturally on blood vessels and when the vessel is damaged or inflamed, the pericyte comes off the vessel and differentiates into an MSC. The newly formed MSC serves the microenvironment and secretes trophic factors such as growth factors, cytokines, chemokines, and extracellular vehicles [157]. These biological characteristics of MSCs support their immunomodulatory effects and regenerative ability [158]. There is no currently available clinical evidence of MSCs in treating long COVID. However, given the unique characteristics of MSCs, some researchers propose that MSC therapies may be beneficial based on the hypothesis that long-COVID is associated with inflammatory processes [159].

Discussion

Long COVID remains a significant medical condition even three years after the initial outbreak, especially with the unknown effect of new variants on its prevalence and severity. Current reports show that COVID-19 survivors have more prevalent anxiety, pain, and depression than those not infected, even two years after the infection [146].

Long COVID typically presents with a cluster of symptoms that affect multiple body systems, including fatigue and dysautonomia, which limit a patient’s ability to participate in daily activities and reduce their quality of life. The convalescence period for long COVID can be lengthy and may require ongoing support which meets with limited availability of post-COVID centers. The discrepancies between the complex symptomatology and lack of guidelines-based treatment can lead to frustration for patients and clinicians. WHO has published a Support for Rehabilitation Self-Management after COVID-19 related illness booklet to help patients guide them towards their recovery. However, there is a continuing need for well-trained specialists and post-COVID clinics to lead treatment in patients with severe long COVID [160].

The treatment can be generally divided into symptoms with medically explicable causes, which should be treated according to guidelines, or those without, which require individual symptom-oriented intervention. Rehabilitation programs provide patients with personalized approaches that include physical therapy, respiratory therapy, occupational therapy, cognitive behavioral therapy, and cardiac rehabilitation, which aim to restore the patient’s previous levels of activity and improve quality of life [161]. Therapy should be directed by the most limiting physical factor and initiated as soon as one month after acute symptom onset if the symptoms persist [60].

Rehabilitation therapies can alleviate dyspnea and breathing abnormalities, improve muscle strength and endurance, promote energy conservation, and facilitate return to activity. Patients with abnormal pulmonary and cardiac function tests may be referred to pulmonary and cardiac rehabilitation which are multidisciplinary programs consisting of monitored progressive aerobic exercise and breathing exercises, energy conservation and pacing, medication management, nutritional guidance, emotional support, and education. Although a systematic review [162] of rehabilitation interventions in long COVID suggested that rehabilitation interventions may be effective in diminishing the long COVID symptoms, further high-quality prospective clinical studies or trials are needed to evaluate the efficacy of rehabilitation therapy in long COVID.

Neurological and neuropsychiatric symptoms in long COVID patients are diverse and include cognitive symptoms such as brain fog, fatigue, myalgia, headaches, sleep disturbance, anxiety, and depression. Following an initial abnormal cognitive screening, a specialist with expertise in formal cognitive assessment and treatment should lead the diagnosis and treatment. Therapeutic approaches for patients with cognitive issues, fatigue, memory, and organization issues should be based on previous knowledge of other post-viral syndromes and brain injury rehabilitation [114]. Multiple treatment approaches should be combined to meet individual patients’ needs. The detrimental effect of sleep disturbances on cognitive functions may be addressed through behavioral sleep hygiene, pharmacological interventions, and treating comorbidities such as sleep apnea.

Fatigue is highly prevalent in long COVID patients, and affects their physical, emotional, and cognitive health [31]. It is important to distinguish between fatigue and diminished activity tolerance as these are related, but distinct conditions with different underlying causes. The fatigue in individuals with long COVID may appear similar to ME/CFS and its treatment is based on ME/CFS recommendations. Therapeutic options require an individualized symptom-guided approach with close monitoring of treatment responses because of potential symptom exacerbation with debilitating post-exertional malaise.

CBT can be an effective approach to address insomnia, brain fog, anxiety, and depression in patients with long COVID. CBT aims to help individuals identify and modify negative thoughts and behavior related to negative symptoms, providing coping strategies to develop a more positive outlook on their recovery. However, it is important to note that CBT may not be effective for everyone, and a qualified mental health professional should be included in the healthcare team to determine the therapy’s benefit.

Regardless of the expected decrease in the severity of COVID-19 disease due to vaccination, there is an emerging question of the evolution of viral pathogenicity. Although most viral changes have little or no impact on the virus’s properties, it is essential to monitor the variants for preparation for future phases of infection. Despite the general expectation of attenuation in virus pathogenicity, a recent study of long-term infection in an immunosuppressed patient from South Africa suggests that future variants may be more pathogenic than the currently circulating Omicron strains [163]. This highlights the importance of research, guidelines for vaccine development, and government vaccination strategies to provide protection for the population. Lastly, while vaccination may decrease the prevalence and severity of COVID-19, there is inadequate data to demonstrate whether or how much effect it has on long COVID [164].

Limitations

The first limitation of our review is the lack of a unified consensus on diagnostic criteria and nomenclature. Long COVID remains a diagnosis of exclusion which introduces variability in the study population and may lead to misdiagnosis of long COVID patients. Secondly, reporting bias stems from the use of electronic health data used in retrospective studies which are more available for hospitalized patients which leads to probable overdiagnosis of respiratory presentation after severe COVID-19. On the other hand, the majority of the current research on mild to moderate COVID-19 relies on self-reported symptoms resulting in reporting bias. The beginning of the pandemic was especially marked by a shortage of diagnostic tests, leaving a large portion of patients with clinical diagnoses and possible misplacement in the study group. The aforementioned reasons have led to variability in reported prevalence rates and difficulties in study comparison. In addition, early studies were conducted on different COVID-19 variants which leads to a limited relevance to the currently circulating virus.

Overall, while the current research on long COVID provides valuable insight into the disease, more well-designed studies and clinical trials are needed to fully understand its prevalence, pathophysiology, identify the specific symptom clusters, and devise an effective treatment.

## Conclusions

Long COVID still requires the attention of the medical community even three years after the pandemic outbreak. Despite not sharing the potentially serious outcomes with acute COVID-19, long COVID significantly decreases quality of life. Its variable manifestations with unclear pathophysiology makes it particularly hard to treat and usually requires a prolonged convalescence and multidisciplinary therapeutic approach. Treatment of patients with long COVID is mostly symptomatic and dictated by the dominant symptom. Symptoms explicable by a well-known medical cause are treated according to current guidelines. Symptoms without any objective final diagnosis remain prevalent and can be addressed by rehabilitation therapy which can help restore patients’ pre-COVID-19 functioning. Chronic fatigue and mental fogginess can respond to cognitive occupational therapy. In summary, long COVID remains a dynamic disease with evolving therapeutic approaches and the need for well-defined treatment guidelines based on well-designed studies.
